# Efficacy and Safety of Insulin Degludec versus Insulin Glargine: A Systematic Review and Meta-Analysis of Fifteen Clinical Trials

**DOI:** 10.1155/2018/8726046

**Published:** 2018-03-12

**Authors:** Wei Liu, Xiaojie Yang, Jing Huang

**Affiliations:** ^1^Department of Cardiology, Guizhou Provincial People's Hospital, Guiyang, China; ^2^Department of Endocrinology, Guizhou Provincial People's Hospital, Guiyang, China

## Abstract

**Aims:**

Insulin degludec (IDeg) and insulin glargine (IGlar) are both proved to be effective in diabetes. This study aimed to assess the effects and safety of IDeg versus IGlar.

**Methods:**

A systematic literature search was conducted using the PubMed, EMBASE, and Cochrane Library electronic databases to identify all randomized controlled trials (RCTs).

**Results:**

Fifteen RCTs were identified. The combined data showed that the decrease in the glycosylated hemoglobin (HbA1c) level was slightly different, and the proportion of patients who achieved HbA1c < 7% was similar between the IDeg and IGlar groups. Further, a statistically significant decrease in the fasting plasma glucose level was observed in the IDeg group as compared to the IGlar group. In patients with T2DM, IDeg was associated with lower rates of overall hypoglycemia. Nocturnal hypoglycemia was significantly lower in the case of IDeg than in the case of IGlar in both T1DM and T2DM patients. No statistically significant differences were observed between the groups.

**Conclusions:**

Compared with IGlar, IDeg is associated with equivalent glycemic control and a statistically significantly lower rate of nocturnal hypoglycemia in patients with T1DM and T2DM. In T2DM patients, IDeg also provides better results in terms of overall hypoglycemia.

## 1. Introduction

Diabetes mellitus (DM) is a major risk factor for cardiovascular diseases and stroke. Improved glycemic control delays and prevents the development of these complications [1]. Unfortunately, a large number of people with DM are unable to achieve the guideline-recommended glycosylated hemoglobin (HbA1c) level [2, 3]. Tighter glycemic control is typically associated with an increased risk of hypoglycemia [4]. The burden and fear of hypoglycemia have become major barriers to patients reaching the recommended HbA1c level [5].

Insulin glargine (IGlar) has been proven to pose a lower risk of hypoglycemia than older human insulin formulations [6]. Nonetheless, the occurrence of hypoglycemia with IGlar treatment is still noticeable [7]. Insulin degludec (IDeg) is a new basal insulin drug with an ultralong duration of action. Experimental studies show that IDeg has a long half-life with a flatter and more stable glucose-lowering effect, resulting in a four times lower within-patient variability than IGlar [8, 9]. Therefore, many randomized controlled trials (RCTs) have been carried out to evaluate the effect of glycemic control and the prevalence of hypoglycemia in the case of IDeg as compared to IGlar [10–14]. A previously published meta-analysis of seven clinical trials showed that IDeg is associated with equivalent HbA1c control and a significantly lower nocturnal hypoglycemia rate than IGlar [15]. However, since then, several new clinical trials with different results have been reported, particularly with respect to nocturnal hypoglycemia; for instance, trials conducted by Pan et al. and Warren et al. showed neutral results [16, 17], while trials from Wysham et al. showed better result for IDeg [18]. Therefore, a systematic review and meta-analysis with updated data are necessary to further assess the efficacy and safety of IDeg compared to that of IGlar.

## 2. Methods

### 2.1. Search Strategy

The PubMed, EMBASE, and Cochrane Library electronic databases were searched for studies published up to July 15, 2017, to identify all publications that compare the effects of the administration of IDeg and that of IGlar in patients with DM. The following terms were used in combination with appropriate logical connectors: “insulin,” “degludec,” “IDeg,” “glargine,” “IGlar,” “randomized,” “randomly,” “diabetes,” and “diabetes mellitus.” Further, a manual search was performed by scanning the references of the identified articles to find studies that were potentially missed by the electronic searches.

### 2.2. Study Selection and Data Collection

The inclusion criteria of the present systematic review and meta-analysis were as follows: (1) an RCT with a no less than 12-week follow-up, (2) patients diagnosed with type 1 DM (T1DM) or type 2 DM (T2DM), and (3) studies that compared the effects of the administration of IDeg once a day with those of IGlar treatment. The exclusion criteria were as follows: (1) IDeg coformulated with other hypoglycemic agents, (2) IDeg injected three times a week, (3) trials with a duration of less than 12 weeks, and (4) short reports, letters to editors, abstracts, or proceedings of scientific meetings.

The study selection was strictly in compliance with the inclusion and exclusion criteria. Two authors (Wei Liu and Xiaojie Yang) independently assessed all the potentially relevant studies. The selection process was carried out by crude screening to exclude a majority of the irrelevant studies at the level of title and abstract, and the remaining studies were double-examined by perusing through the full text to reach the final decision. A consensus was reached on all eligible studies between the two screening authors. Any discrepancies were resolved by discussion.

Two authors (Wei Liu and Jing Huang) independently extracted all the relevant information from the eligible studies. A prespecified table that contained the relevant items was used to help with the data collection.

### 2.3. Endpoints

The treatment efficacy was evaluated on the basis of the change in the HbA1c and fasting plasma glucose (FPG) levels from the baseline to the end of the study and the proportion of patients who achieved HbA1c levels of <7%. The safety assessments considered adverse events, hypoglycemia, and body weight. The same hypoglycemia criteria were used for all the included studies in our manuscript. Hypoglycemia was defined as a symptomatic or an asymptomatic event with plasma glucose of <3.1 mmol/L (56 mg/dL) [13].

### 2.4. Evaluation of Study Quality and Publication Bias

The quality of the included studies was evaluated by using the Jadad scale. The Jadad scale consists of three items pertaining to the descriptions of randomization (0–2 points), double blinding (0–2 points), and dropouts and withdrawals (0-1 point), totaling to five points, with a higher score indicating better quality. Trials that scored 3 points or more were considered to be high-quality trials.

Publication bias was evaluated by using a funnel plot and visually inspecting its symmetry.

### 2.5. Data Synthesis and Statistical Analysis

The *I*^2^ statistic was used to test statistical heterogeneity, with values of >50% representing important heterogeneity, then a random-effects model was used to perform the meta-analysis. The mean difference (MD) with the 95% confidence interval (CI) between the IDeg and the IGlar cases was calculated to represent the difference in the changes in the HbA1c and FPG levels. For the efficacy analysis, the odds ratio (OR) or the risk ratio (RR) was calculated as the effect size. A subgroup analysis was performed between patients with T1DM and those with T2DM.

The present systematic review and meta-analysis were performed in compliance with the recommendations of the Preferred Reporting Items for Systematic Reviews and Meta-Analyses (PRISMA) [19]. All meta-analyses of the present study were pooled according to *Cochrane Handbook for Systematic Reviews of Interventions Version 5.1.0*. The statistical significance was set at *p* < 0.05. All analyses were conducted using the software Review Manager 5.3.

## 3. Results

Finally, 15 studies were found to be eligible for this research [10–14, 16–18, 20–26]. All the included studies were multicenter studies, except the study by Iga et al., which was a single-center study [25]. The duration of intervention ranged from 12 weeks to 2 years. Four of these studies used a crossover design [17, 18, 25, 26]. All 15 studies had a so-called treat-to-target design.

In all, 16,328 patients were included in the present study. Five studies recruited patients with T1DM [11, 14, 22, 25, 26], and the other ten studies enrolled patients with T2DM [10, 12, 13, 16–18, 20, 21, 23, 24]. In all the considered studies, the authors used an intention-to-treat analysis. Withdrawals and dropouts were described adequately in all these studies, and the rates of completed treatment varied from 76.7% to 100%. The clinical characteristics of each trial are summarized in Table 1.

### 3.1. Glycemic Control

**Figure 1:**
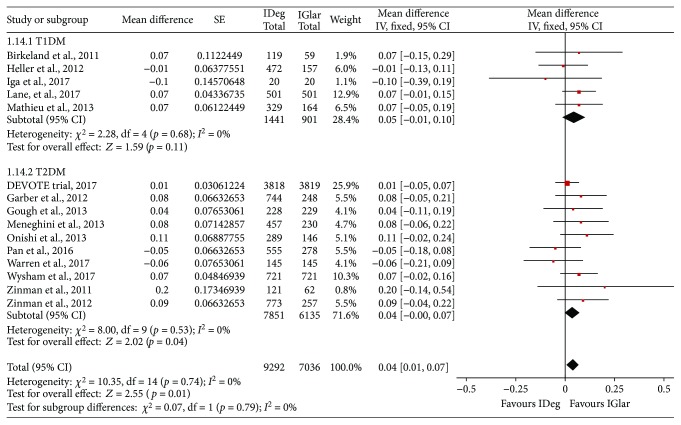
Mean difference in the changes in the glycosylated hemoglobin (HbA1c) level between the IDeg and IGlar groups: IDeg: insulin degludec; IGlar: insulin glargine; T1DM: type 1 diabetes mellitus; T2DM: type 2 diabetes mellitus; CI: confidence interval; IV: inverse variance.

**Figure 2:**
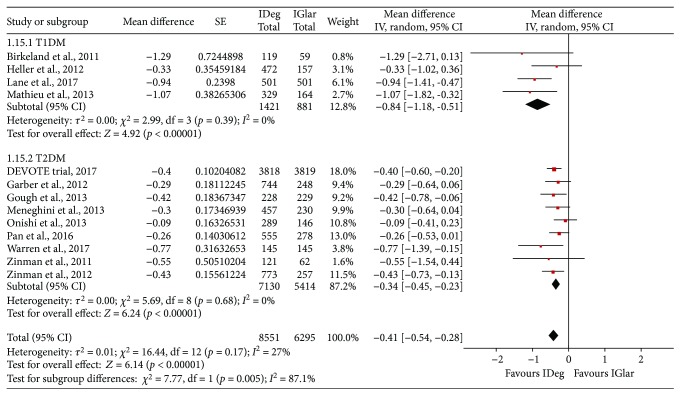
Mean difference in the changes in the fasting plasma glucose (FPG) level between the IDeg and the IGlar groups: the abbreviations are the same as Figure 1.

The HbA1c and the changes from the baseline to the endpoint levels were reported in all the 15 included studies. The overall meta-analysis revealed statistically significant difference with a MD of 0.04% in the HbA1c level between the IDeg and the IGlar treatment groups, with nonsignificant heterogeneity (MD = 0.04%, 95% CI = 0.01% to 0.07%, *p* = 0.01, *I*^2^ = 0%, Figure 1 and Table 2). The subgroup analyses showed nonsignificant difference in T1DM (MD = 0.05%, 95% CI = −0.01% to 0.10%, *p* = 0.11, *I*^2^ = 0%) and slight difference in T2DM (MD = 0.04%, 95% CI = 0.00 to 0.07%, *p* = 0.04, *I*^2^ = 0%). In the case of FPG level, the IDeg treatment was associated with a statistically significant reduction as compared to the IGlar treatment (MD = −0.41, 95% CI = −0.54 to −0.28, *p* < 0.001, *I*^2^ = 27%, Figure 2 and Table 2); this association was observed in the cases of both T1DM (MD = −0.84, 95% CI = −1.18 to −0.51, *p* < 0.001, *I*^2^ = 0%) and T2DM (MD = −0.34, 95% CI = −0.45 to −0.23, *p* < 0.001, *I*^2^ = 0%).

Eight studies reported the following proportions of patients who achieved HbA1c levels of <7%: 1704 (46.1%) of 3693 patients achieved HbA1c < 7% in the IDeg group, and 793 (46.9%) of 1690 patients achieved HbA1c < 7% in the IGlar group. The meta-analysis showed that the proportions of patients who achieved HbA1c levels of <7% were similar in the two groups (*p* = 0.19, Table 2).

### 3.2. Safety Endpoints

All 15 included studies evaluated the changes in body weight. The pooled result showed a similar change in body weight between the examined groups (MD = 0.03, 95% CI = −0.11 to 0.18, *p* = 0.67, *I*^2^ = 36%, Table 2), of both patients with T1DM (MD = −0.04, 95% CI = −0.35 to 0.26, *p* = 0.78, *I*^2^ = 0%) and those with T2DM (MD = 0.05, 95% CI = −0.11 to 0.22, *p* = 0.52, *I*^2^ = 51%).

**Figure 3:**
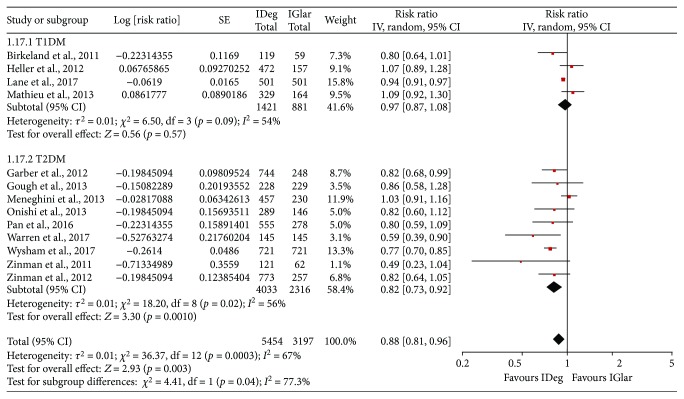
Comparison of the risk of overall hypoglycemia (events per patient-year of episode) between IDeg and IGlar across subgroups: the abbreviations are the same as Figure 1.

With respect to overall hypoglycemia, events per patient-year of exposure were integrated. We identified 13 studies that reported the events per patient-year of overall hypoglycemia. The meta-analyses showed that the incidence of overall hypoglycemia was lower in the IDeg treatment group (RR = 0.88, 95% CI = 0.81 to 0.96, *p* = 0.003, *I*^2^ = 67%, random-effects model, Figure 3 and Table 2). Subgroup analyses revealed that IDeg reduced overall hypoglycemia only in patients with T2DM (RR = 0.82, 95% CI = 0.73 to 0.92, *p* = 0.001, *I*^2^ = 56%) and not in patients with T1DM.

**Figure 4:**
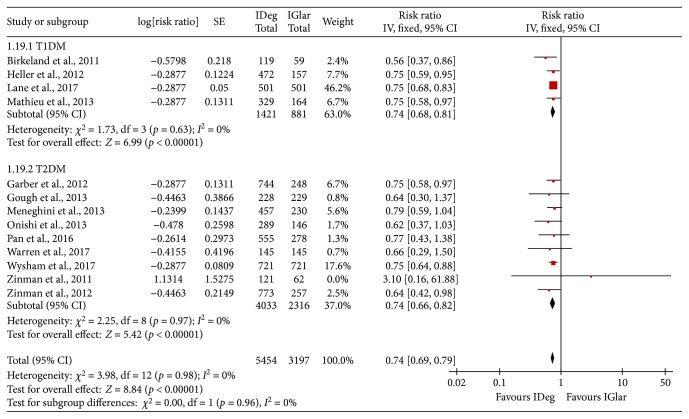
Comparison of the risk of nocturnal hypoglycemia (events per patient-year of episode) between IDeg and IGlar across subgroups: the abbreviations are the same as Figure 1.

With respect to nocturnal hypoglycemia, events per patient-year of the episodes were lower in the IDeg group in the cases of both T1DM and T2DM (overall analysis: RR = 0.74, 95% CI = 0.69 to 0.79, *p* < 0.001, *I*^2^ = 0%; T1DM: RR = 0.74, 95% CI = 0.68 to 0.81, *p* < 0.001, *I*^2^ = 0%; T2DM: RR = 0.74, 95% CI = 0.66 to 0.82, *p* < 0.001, *I*^2^ = 0%, Figure 4 and Table 2).

### 3.3. Adverse Events

Twelve studies reported the adverse events in detail. The proportion of patients reporting adverse events did not differ between the groups (4785 [53.7%] of the 8911 patients in the IDeg group versus 3336 [49.7%] of the 6715 patients in the IGlar group, pooled OR = 0.94, 95% CI = 0.88 to 1.01, *p* = 0.09, *I*^2^ = 0%, Table 2), both for patients with T1DM and for those with T2DM. Of the three studies that did not report the adverse events in detail, one study did not report any adverse events [25], and the remaining two studies reported that the rates of adverse events were comparable between the groups [11, 17].

No statistically significant differences were observed between the groups for the serious adverse events and the adverse events possibly related to the trial product (Table 2).

### 3.4. Quality and Publication Bias of the Included Studies

The quality of the included studies was quantitatively assessed by using the Jadad scale. 14 of the 15 included studies were multicenter designs. Further, all the studies had Jadad scores of 3 points or more. Therefore, all the included studies were of high quality (Table 3). Publication bias was determined on the basis of the asymmetrical funnel plots.

## 4. Discussion

The present systematic review and meta-analysis included 15 high-quality RCTs to evaluate the efficacy and safety of two long-acting insulin analogs. The pooled results demonstrated the following: (1) although the IDeg treatment achieved a significantly better result than the IGlar treatment in terms of the FPG level, the reduction of HbA1c was comparable between the IDeg and the IGlar treatments. The results were robust across the T1DM and T2DM subgroups. (2) The risk of hypoglycemia was statistically significantly decreased in the IDeg treatment group as compared to that in the IGlar treatment group of patients with T2DM, as was nocturnal hypoglycemia. While in patients with T1DM, the IDeg treatment was associated with a lower risk of nocturnal hypoglycemia, but not associated with a lower risk of overall hypoglycemia. (3) The adverse events and the serious adverse events were similar between the IDeg and the IGlar treatment groups and across the T1DM and T2DM subgroups.

Glycemic control is vital for patients with DM. The microvascular and macrovascular complications of DM declined dramatically over the past two decades [27] but have reappeared with a higher rate of hospital admissions for hypoglycemic events [7]. Therefore, the development of an effective antidiabetic treatment with a lower rate of hypoglycemic events than that with the current treatment is important.

The present study showed that the IDeg treatment exhibited a slight increase of HbA1c to that observed in the case of IGlar treatment with no clinical significant effect, followed with a greater decrease in the FPG level. Therefore, we inferred that IDeg is noninferior to IGlar with respect to glycemic control. Similar efficacy is expected because all the included studies were treat-to-target trials, and noninferiority was observed in each trial. This finding is of great importance to confirm that the lower rates of hypoglycemia observed in the case of the IDeg treatment are not achieved at the cost of poor glycemic control.

Hypoglycemia is a common complication of insulin treatment in patients with DM [7]. Hypoglycemia has been considered to be one of the main barriers to good glycemic control, resulting in patients becoming unwilling to optimize treatment with insulin and in clinicians conservatively recommending more aggressive treatment targets [28]. In the present study, we found that the IDeg treatment was associated with a significant reduction in the risk of hypoglycemia, particularly nocturnal hypoglycemia. Besides, several studies showed that IDeg treatment not only decreased the risk of hypoglycemia but also led to improvements in both mental and physical health status [12, 13].

In patients with T2DM, the rate of nocturnal hypoglycemia decreased significantly along with a reduction in overall hypoglycemia. Although a previous meta-analysis reported that IDeg did not decrease the rate of overall hypoglycemia [15], the present meta-analysis including more recent studies showed a statistically significant decline in hypoglycemia. With respect to nocturnal hypoglycemia, a more statistically significant effect was observed than that for overall hypoglycemia in the case of the IDeg treatment.

In patients with T1DM, the IDeg treatment was associated with a reduction in nocturnal hypoglycemia, but not in overall hypoglycemia. Concerns about the increase in the rate of daytime hypoglycemia were raised. However, nocturnal hypoglycemia poses a considerably greater risk to patients with DM than daytime hypoglycemia [29]. Therefore, IDeg is still a safer basal insulin option than IGlar. Nonetheless, only four studies evaluated the hypoglycemia rate in the T1DM subgroup; therefore, the explanation of the results should be cautious.

It is striking that despite the lower rates of hypoglycemia, glycemic control was not evidently compensated. The present meta-analysis showed that IDeg exhibited significantly better results than IGlar in terms of the FPG level and the hypoglycemia rate, with similar reductions in the HbA1c level. Lower FPG values are typically expected to be followed by higher rates of nocturnal hypoglycemia, but in the case of IDeg, the results were the opposite. This difference in results could be attributed to the fact that IDeg has a stable and consistent glucose-lowering effect, with its ultralong duration of action and lower within-patient day-to-day variability than IGlar [8, 9].

Although our study included 15 high-quality RCTs, it has some important limitations. Firstly, most of the included studies had self-reporting of hypoglycemic episodes; this posed a potential risk of failure in the reporting of the episodes. Secondly, hypoglycemia has different definitions across the European Medicines Agency and American Diabetes Association [30]. However, in the present systematic review and meta-analysis, only a symptomatic or an asymptomatic event with plasma glucose of <3.1 mmol/L (56 mg/dL) was defined as hypoglycemia. Therefore, the effects of hypoglycemia were not covered by other definitions, and the extrapolation of the results should be cautious to other definitions of hyperglycemia. Nonetheless, a previous meta-analysis reported similar results for hypoglycemia defined differently [30]. Thirdly, the inherent limitations of a meta-analysis cannot be ignored, such as publication bias.

In conclusion, this systematic review and meta-analysis of 15 RCTs demonstrate that IDeg exhibits a similar reduction of HbA1c to that of IGlar but a lower FPG value. The rates of nocturnal hypoglycemia were significantly decreased in the IDeg group for both T1DM and T2DM patients, while the overall hypoglycemia was only reduced in patients with T2DM. These findings indicate that IDeg might be a safer option to patients with diabetes mellitus who need basal insulin therapy.

## Figures and Tables

**Table 1:** Baseline characteristics of the included studies.

Study, year	Patients	Sample size		Age (y)		Male (%)		Duration of DM (y)		HbA1c (%)		Trial duration (weeks)
		IDeg	IGlar	IDeg	IGlar	IDeg	IGlar	IDeg	IGlar	IDeg	IGlar	
Zinman et al., 2011^∗^ [10]	T2DM	121	62	55.3 (8.7)	53.9 (8.5)	60%	60%	7.2 (4.5)	6.7 (5.0)	8.7 (1.2)	8.7 (1.1)	16
Birkeland et al., 2011^∗^ [11]	T1DM	119	59	44.5 (12.7)	45.6 (12.5)	62%	54%	21.7 (11.8)	19.1 (10.8)	8.5 (1.0)	8.3 (0.8)	16
Zinman et al., 2012 [12]	T2DM	773	257	59.3 (9.7)	58.7 (9.9)	61%	65%	9.4 (6.3)	8.6 (5.7)	8.2 (0.8)	8.2 (0.8)	52 (+52^ex^)
Garber et al., 2012 [13]	T2DM	744	248	59.2 (9.1)	58.1 (10.0)	54%	54%	13.6 (7.4)	13.4 (6.9)	8.3 (0.8)	8.4 (0.9)	52 (+26^ex^)
Heller et al., 2012 [14]	T1DM	472	157	42.8 (13.7)	43.7 (13.3)	59%	57%	19.1 (12.2)	18.2 (11.4)	7.7 (0.9)	7.7 (1.0)	52 (+52^ex^)
Onishi et al., 2013 [20]	T2DM	289	146	58.8 (9.8)	58.1 (10.1)	55%	51%	11.8 (6.5)	11.1 (6.5)	8.4 (0.8)	8.5 (0.8)	26
Gough et al., 2013 [21]	T2DM	228	229	57.8 (9.0)	57.3 (9.4)	52%	54%	8.4 (6.7)	8.0 (5.6)	8.3 (1.0)	8.2 (0.9)	26
Mathieu et al., 2013^∗^ [22]	T1DM	329	164	43.6 (13.1)	44.1 (12.6)	60%	54%	18.7 (12.5)	18.2 (11.9)	7.7 (1.0)	7.7 (0.9)	26 (+26^ex^)
Meneghini et al., 2013^∗^ [23]	T2DM	457	230	56.3 (10.1)	56.7 (8.8)	57%	48%	10.6 (6.8)	10.8 (6.4)	8.5 (1.0)	8.4 (0.9)	26
Pan et al., 2016 [16]	T2DM	555	278	55.9 (9.7)	56.6 (9.2)	54%	47%	7.6 (5.3)	8.3 (5.5)	8.3 (0.9)	8.3 (0.8)	26
DEVOTE trial, 2017 [24]	T2DM	3818	3819	64.9 (7.3)	65.0 (7.5)	63%	62%	16.6 (8.8)	16.2 (8.9)	8.4 (1.6)	8.4 (1.7)	108
Iga et al., 2017^#^ [25]	T1DM	20	20	55 (14)	53 (18)	50%	60%	14.4 (8.6)	16.1 (8.7)	7.1 (0.9)	7.7 (0.6)	12
Warren et al., 2017^#^ [17]	T2DM	145	145	54.7 (10.2)	55.8 (9.0)	58%	67%	12.1 (6.7)	12.1 (7.9)	8.0 (1.1)	8.3 (1.4)	16
Lane et al., 2017^#^ [26]	T1DM	501	501	45.4 (13.7)	46.4 (14.6)	51%	57%	23.2 (13.5)	23.6 (13.4)	7.7 (1.0)	7.5 (1.0)	32
Wysham et al., 2017^#^ [18]	T2DM	721	721	61.5 (10.7)	61.2 (10.3)	53%	53%	14.2 (8.3)	13.9 (8.0)	7.6 (1.1)	7.6 (1.1)	32

Data are shown as numbers or means (standard deviation) unless otherwise stated. IDeg: insulin degludec; IGlar: insulin glargine; T1DM: type 1 diabetes mellitus; T2DM: type 2 diabetes mellitus; HbA1C: hemoglobin A1c. ^#^They were crossover trials; data were from the first period. ^∗^These studies had two intervention groups. Groups were combined by formulae from *Cochrane Handbook for Systematic Reviews of Interventions (Version 5.1.0).* ex: extension of trial.

**Table 2:** Summary of the main results.

Population	N1	T1DM: pooled effect size IDeg : IGlar (95% confidence interval)	N2	T2DM: pooled effect size IDeg : IGlar (95% confidence interval)	*N* (N1 + N2)	Overall: pooled effect size IDeg : IGlar (95% confidence interval)
Items						
Change of HbA1c	2342	MD = 0.05% (−0.01%, 0.10%)	13,986	MD = 0.04% (0.00, 0.07%)^#^	16,328	MD = 0.04% (0.01%, 0.07%)^#^
Change of FPG	2302	MD = −0.84 (−1.18, −0.51)^#^	12,544	MD = −0.34 (−0.45, −0.23)^#^	14,846	MD = −0.41 (−0.54, −0.28)^#^
Change of body weight	2342	MD = −0.04 (−0.35, 0.26)	13,986	MD = 0.05 (−0.11, 0.22)	16,328	MD = 0.03 (−0.11, 0.18)
Participants achieved HbA1c levels of <7%	629	RR = 0.93, (0.75, 1.15)	4754	RR = 0.96, (0.90, 1.03)	5383	RR = 0.96, (0.90, 1.02)
Overall hypoglycemia	2302	RR = 0.97 (0.87, 1.08)	6349	RR = 0.82 (0.73, 0.92)^#^	8651	RR = 0.88 (0.81, 0.96)^#^
Nocturnal hypoglycemia	2302	RR = 0.74 (0.68, 0.81)^#^	6349	RR = 0.74 (0.66, 0.82)^#^	8651	RR = 0.74 (0.69, 0.79)^#^
Adverse events	2036	OR = 0.96 (0.78, 1.19)	13,590	OR = 0.94 (0.87, 1.01)	15,626	OR = 0.94 (0.88, 1.01)
Serious adverse events	2214	OR = 0.89 (0.67, 1.18)	13,590	OR = 0.95, (0.87, 1.03)	15,804	OR = 0.95 (0.87, 1.02)
Adverse events possibly/probably related to the trial product	2036	OR = 1.24 (0.93, 1.64)	5496	OR = 1.05 (0.86, 1.29)	7532	OR = 1.11 (0.94, 1.31)

IDeg: insulin degludec; IGlar: insulin glargine; N1: number of patients with T1DM; N2: number of patients with T2DM; *N*: number of patients; T1DM: type 1 diabetes mellitus; T2DM: type 2 diabetes mellitus; MD: mean difference; RR: risk ratio; OR: odds ratio. ^#^*p* < 0.05.

**Table 3:** The design and quality assessment of individual study.

Study, year	Study design	Descriptions of randomization	Double blinding	Dropouts and withdrawals	Jadad score^∗^
Zinman et al., 2011 [10]	Multicenter, parallel group trial	2	0	1	3
Birkeland et al., 2011 [11]	Multicenter, parallel group trial	2	0	1	3
Zinman et al., 2012 [12]	Multicenter, parallel group trial	2	0	1	3
Garber et al., 2012 [13]	Multicenter, parallel group trial	2	0	1	3
Heller et al., 2012 [14]	Multicenter, parallel group trial	2	0	1	3
Onishi et al., 2013 [20]	Multicenter, parallel group trial	2	0	1	3
Gough et al., 2013 [21]	Multicenter, parallel group trial	2	0	1	3
Mathieu et al., 2013 [22]	Multicenter, parallel group trial	2	0	1	3
Meneghini et al., 2013 [23]	Multicenter, parallel group trial	2	0	1	3
Pan et al., 2016 [16]	Multicenter, parallel group trial	2	0	1	3
DEVOTE trial, 2017 [24]	Multicenter, parallel group trial	2	2	1	5
Iga et al., 2017 [25]	Single-center, crossover trial	2	0	1	3
Warren et al., 2017 [17]	Multicenter, crossover trial	2	0	1	3
Lane et al., 2017 [26]	Multicenter, crossover trial	2	2	1	5
Wysham et al., 2017 [18]	Multicenter, crossover trial	2	2	1	5

^∗^The Jadad scale consists of three items related to descriptions of randomization (0–2 points), double blinding (0–2 points), and dropouts and withdrawals (0-1 point) for a total of five scores. Higher scores indicate better quality. High-quality trials were defined as those that scored more than 2. Low-quality trials were defined as those that scored 2 or less.
